# Transcatheter Removal of Embolized Port Catheters from the Hearts of Two Children

**DOI:** 10.1155/2015/973196

**Published:** 2015-04-29

**Authors:** Osman Baspinar, Ayse Sulu, Derya Aydin Sahin

**Affiliations:** Department of Pediatric Cardiology, Gaziantep University Medical Faculty, 27310 Gaziantep, Turkey

## Abstract

Embolization of a port catheter is a dangerous and serious complication. In this paper, we present two cases of children, aged 4.5 months and 6 years, in whom port catheters had embolized to the right ventricle one month and 1.5 years priorly, respectively; the port catheters were retrieved via snaring.

## 1. Introduction

The placement of port catheters offers great advantages to patients who require long-term intravenous treatment. Like many medical interventions, this procedure has inherent dangers as well as potentially complicated side effects and risks. One such complication is embolization of the port catheter, a situation that has significant risks to the patient [1, 2]. In the patients examined in this study, we were able to successfully retrieve embolized port catheters using a transcatheter approach, despite the relatively long time periods of time that had elapsed following the embolization events (1 month and 1.5 years, resp.).

## 2. Case 1

**Figure 1:**
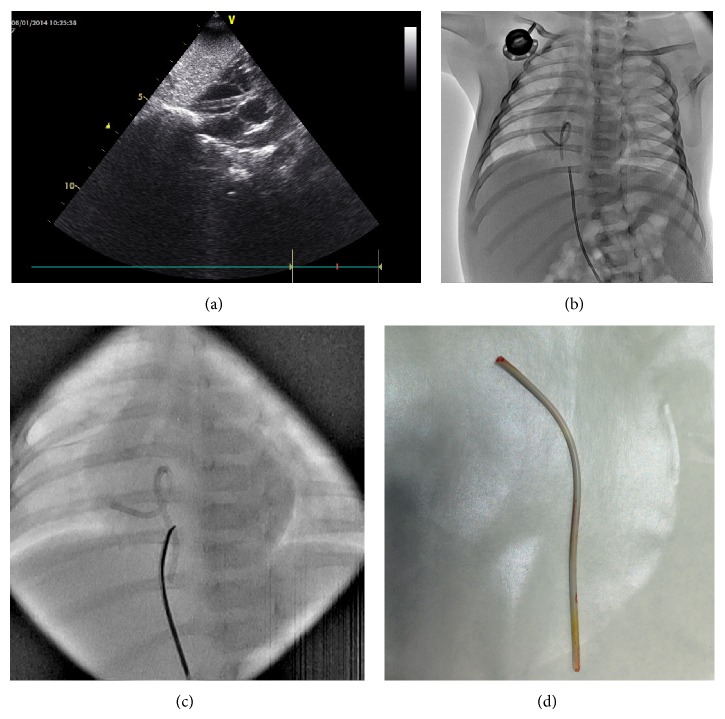
(a) Echocardiographic and (b) angiographic images of an embolized catheter making a loop in the heart of a 4.5-month-old infant and (c) images of its retrieval via snare are shown. (d) The embolized port catheters.

A 4.5-month-old male infant with a diagnosis of acute lymphoblastic leukemia was receiving chemotherapy. A port catheter was placed into his right subclavian vein one month priorly. On routine chest X-ray, it was observed that the catheter was separated from the port chamber along the entirety of its length and that it had fallen into the right atrium. Transthoracic echocardiography revealed that the distal end of the catheter was in the inferior vena cava. The distal end had entered the right ventricle via the tricuspid valve and, after making a loop, reentered the right atrium. Cardiac catheterization was performed by entry through the left femoral vein, which enabled the embolized catheter to be grasped in the inferior vena cava from its distal end via a 10 mm Amplatz gooseneck snare tip (Plymouth, MN, USA); it was removed easily and quickly (Figures 1(a)–1(d)). The duration of the fluoroscopy was 1.7 minutes. The catheter measured 8 cm in length, and there were no complications. One month later, the original catheter port chamber was surgically removed and a new port system was put into place.

## 3. Case 2

**Figure 2:**
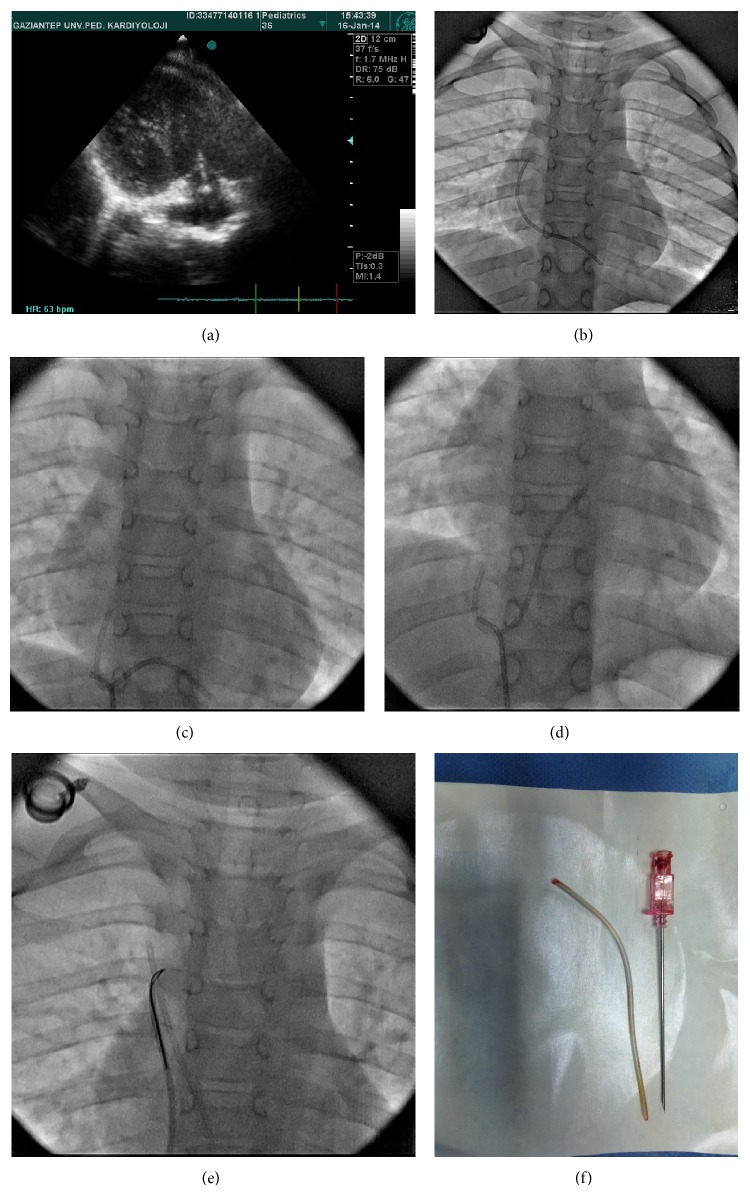
(a) Echocardiographic and (b) angiographic images of a catheter extending to the right ventricle apex from the right atrium roof in a 6-year-old child. ((c), (d), and (e)) Images of its retrieval using a Judkins right catheter as a hook to snare the catheter are shown. (f) The embolized port catheters.

A routine chest X-ray that was performed on a 6-year-old asymptomatic patient with acute lymphocytic leukemia in remission revealed that a port catheter that was placed in the patient's subclavian vein had detached from the port chamber and embolized. When the previous X-ray films were checked, it was found that the right-sided port catheter could be observed in the right ventricle in a film that was taken 1.5 years priorly. Additionally, there was significant tricuspid insufficiency which was noted by transthoracic echocardiography. During cardiac catheterization, the catheter could not be grasped using a 10 mm Amplatz gooseneck snare tip (Plymouth, MN, USA) because it was extending into the apex of the right ventricle from the roof of the right atrium. Instead, using a Judkins right catheter, it was hooked across the middle and pulled back into the inferior vena cava. The displaced catheter shifted to the superior vena cava and was then grasped from the proximal end via the same snare and retrieved (Figures 2(a)–2(f)). The duration of fluoroscopy was 4.8 minutes. The port chamber was surgically removed, as there was no longer a need for chemotherapy.

## 4. Discussion

Venous catheters are important devices that are used for transvenous fluid management and chemotherapeutic drug administration. They provide great comfort for pediatric patients by preventing recurrent intravenous line placement; however, as with many interventions, the use of a port system has its own complications, including port catheter embolization. This embolization is a rare and serious complication that is caused either by the compression of the catheter between the clavicle and costa, called pinch-off, or by the detachment of the catheter from the portal directly [1, 2]. Catheter fragments primarily embolize to the pulmonary artery and right ventricle, and the embolized fragments can remain in the heart for a long time without causing any symptoms [3]; however, they should be removed after diagnosis to prevent symptoms from developing. Frequently, embolized catheter fragments are not detected for prolonged periods of time; generally, they are found incidentally. Indeed, Surov et al. [3] discovered that these embolisms were found incidentally in over fifty percent of the patients that have them. In the patients studied here, the embolized catheters were also asymptomatic, although it should be noted that another meta-analysis found that only 24.2% of cases were asymptomatic; it is even more concerning that the mortality rate was 1.8% [4].

In both of our patients, because the integrity of the catheter was not compromised, the reason for catheter displacement was likely a faulty connection between the catheter and the port chamber. Additionally, after the surgical procedures, it was confirmed that, in both cases, the catheter parts were totally detached from the port chamber. Thus, patient's chest X-rays should be regularly inspected after port catheter placement to assess the risk of embolization, especially in case of port catheter malfunction. However, if embolization does occur, it should be remembered before the need for cardiac surgery is decided upon that catheters can be retrieved using a transcatheter approach in experienced medical centers. As shown in our patients, it is possible to quickly and safely retrieve the catheters from patients in the pediatric age group using a snare catheter via a transcatheter approach.
